# Search Techniques for the Web of Things: A Taxonomy and Survey

**DOI:** 10.3390/s16050600

**Published:** 2016-04-27

**Authors:** Yuchao Zhou, Suparna De, Wei Wang, Klaus Moessner

**Affiliations:** 1 Institute for Communication Systems (ICS), University of Surrey, Guildford GU2 7XH, UK; s.de@surrey.ac.uk (S.D.); k.moessner@surrey.ac.uk (K.M.); 2 Department of Computer Science and Software Engineering, Xi’an Jiaotong-Liverpool University, Ren’ai Road Dushu Lake Higher Education Town SIP, Suzhou 215123, China; Wei.Wang03@xjtlu.edu.cn

**Keywords:** search, Web of Things, Internet of Things, linked data, streaming data, observation and measurement data, sensors, entities

## Abstract

The Web of Things aims to make physical world objects and their data accessible through standard Web technologies to enable intelligent applications and sophisticated data analytics. Due to the amount and heterogeneity of the data, it is challenging to perform data analysis directly; especially when the data is captured from a large number of distributed sources. However, the size and scope of the data can be reduced and narrowed down with search techniques, so that only the most relevant and useful data items are selected according to the application requirements. Search is fundamental to the Web of Things while challenging by nature in this context, e.g., mobility of the objects, opportunistic presence and sensing, continuous data streams with changing spatial and temporal properties, efficient indexing for historical and real time data. The research community has developed numerous techniques and methods to tackle these problems as reported by a large body of literature in the last few years. A comprehensive investigation of the current and past studies is necessary to gain a clear view of the research landscape and to identify promising future directions. This survey reviews the state-of-the-art search methods for the Web of Things, which are classified according to three different viewpoints: basic principles, data/knowledge representation, and contents being searched. Experiences and lessons learned from the existing work and some EU research projects related to Web of Things are discussed, and an outlook to the future research is presented.

## 1. Introduction

The Web of Things (WoT) paradigm envisions an interoperable infrastructure for enabling communications among physical world objects and data access to create future Internet of Things (IoT) applications through existing Web standards [1,2]. In recent years, low price and easy deployment of sensors and wireless sensor networks, the development of communication techniques, and the emergence of various smart objects, have led to increasing numbers of physical objects being connected. Cisco Systems predicts the number of connected objects to increase to 50 billion by 2020 [3]. As a consequence, massive amount of data (e.g., data describing the objects or data captured from the physical or social worlds) is expected to be generated continuously by these connected objects.

In WoT, the physical objects are deployed in different geographical locations and managed by different organisations; and the captured data is represented in different formats and its quality is subject to various uncertainties. Currently, the data is collected and processed by different brokers or middleware, stored in distributed datasets or clouds, and often made available through specialised Application Programming Interfaces (APIs). This implies a key obstacle in developing innovative applications in the WoT, which is, multiple sources of data are required to be blended to detect useful patterns and discover valuable information. As not all data is relevant to an application’s needs and requirements, efficient and effective search methods are needed to find the most relevant and useful data sources (*i.e.*, the sources that produce the data) or data items.

In contrast to searching documents on the Web, search in WoT manifests several additional challenges. For example, WoT objects can have complex descriptions; and their produced data can have properties represented along the thematic, spatial, and temporal dimensions. The status of the WoT objects may change unexpectedly, for instance, loss of communication, malfunctioning of the wireless sensor nodes, opportunistic presence of the mobile sensors, and change of the measurement targets. All of these characteristics indicate that traditional search techniques built on relatively static indexing structures are not sufficient for WoT applications.

A notable research direction is on device and service discovery on the IoT. A number of EU research initiatives have focussed on developing device and service resolution frameworks for the IoT. The IoT-A [4] project provides semantic description models for services and entities to enable scalable lookup and discovery of resources (devices, sensors), real world entities and their associations [5]. IoT@Work [6] also provides a directory service that enables access to services and devices through a variety of protocols and tools. The VITAL project [7] aims to enable semantic discovery of Internet-connected objects and services agnostic of the underlying IoT platform. Research in this line in fact targets searching objects of different types (e.g., devices, sensors, and services) which generate data. There is also research on IoT discovery at the lower layers of the IP stack, focusing on connection and access, for example, neighbour discovery in Wireless Sensor Networks [8] and opportunistic IoT scenarios [9]. Service discovery for constrained machine-to-machine communications is reviewed in [10]. In contrast, this study investigates the search problems on the WoT from a data-centric perspective.

**Figure 1:**
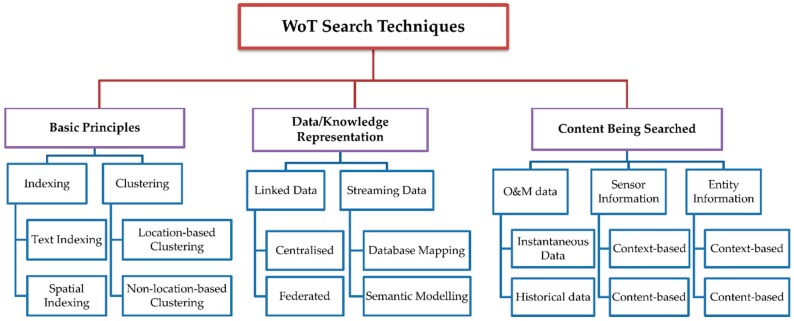
Classifications of the Search Techniques in WoT.

Currently, there are only a limited number of studies that survey the search techniques for the WoT. One such study reviews research in searching for entities [11], however, search for sensors and Observation and Measurement (O&M) data is not included. O&M data represents values of any observed or measured quantity (e.g., indoor temperature). Since sensor networks have been increasingly deployed to monitor environments all around the world, sensors and their O&M data are becoming more and more important in the Web of Things. Nevertheless, in order to utilise sensors and O&M data for different WoT applications, a coherent infrastructure is needed for sharing, finding, and accessing sensors and their O&M data, due to the heterogeneity of protocols supported by the various sensors from a huge number of sensor manufacturers [12]. Formal representations of both sensor model (such as SensorML [13]) and data model (such as Observations and Measurements XML Schema [14]) are required for such an infrastructure. Another survey [2] presents search techniques according to a simple classification on the type of data aggregation (push- or pull-based). It does not provide a clear view of different facets of search techniques. In [15], search systems are compared along multiple dimensions, however, a classification that allows a better understanding of the research is missing. Overall, the existing studies only focus on some specific aspects and are insufficient to offer a holistic and clear view of the state-of-the-art. This paper aims to provide a comprehensive survey which identifies the different perspectives that mirror the development of the search techniques. It defines a taxonomy, shown in Figure 1, that classifies the search techniques for WoT from three perspectives: (1) fundamental search principles; (2) data/knowledge representation, focusing on Linked Data and streaming data; and (3) contents being searched (e.g., Observation and Measurement data, sensors, and entities in the WoT). It should be noted that the techniques classified according to these three perspectives overlap with each other to some extent.

The contributions of this paper can be summarised as follows. (1) A comprehensive, qualitative comparison of different search techniques for the WoT, which enables the readers to gain a clear picture of the current research landscape; (2) a classification based on (a) fundamental search principles, which provides the readers a better understanding of the enabling techniques, (b) data/knowledge representation, which enables the readers to understand how different knowledge representation formalisms contribute to the search in WoT, (c) contents being searched, which enables the readers to understand how the variety of data impacts effective and efficient design of search methods; (3) a critical discussion of the lessons and experiences gained from this study and the authors’ involvement in some of the large EU research projects on WoT and IoT; and (4) observation of the trends and promising future research directions based on the review of the literature.

The remainder of this paper is organised as follows: in Section 2 we provide some background information on the WoT system model and relevant applications. Metrics used for comparing the search techniques are introduced in Section 3. Section 4, Section 5 and Section 6 detail the different techniques that fit into the classification model: basic principles, data/knowledge representation, and content of data, respectively. A critical discussion on the limitations, best practices and opportunities are provided in Section 7. Section 8 gives an outlook for future research and Section 9 concludes the paper.

## 2. WoT System Model and Applications

**Figure 2:**
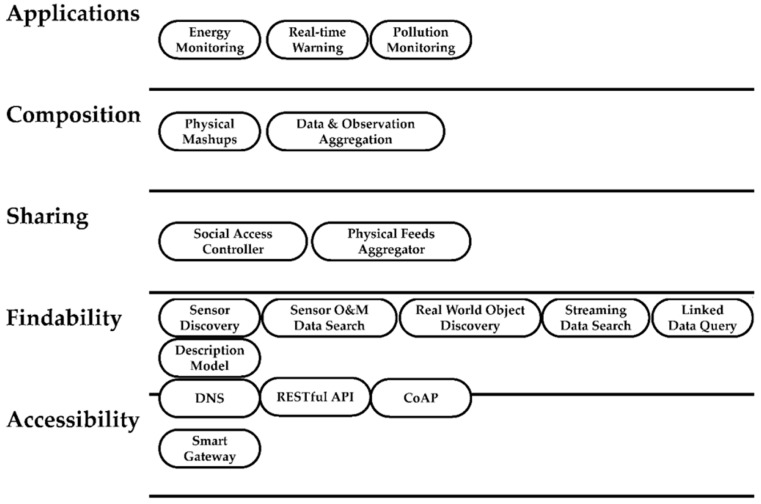
WoT System Model.

The Web of Things provides an Application Layer that simplifies the creation of Internet of Things applications. For this study, we adopt the WoT system model proposed in [16] (shown in Figure 2). Applications can be built based on four other layers, namely, Accessiblity Layer, Findability Layer, Sharing Layer, and Composition Layer. The Accessibility Layer deals with the problem of “how can we, from an application point of view, enable a consistent access to all kinds of connected objects?” [16]. This can be enabled by providing Smart Gateway, RESTful API [17], as well as the Domain Name System (DNS) [18] and Constrained Application Protocol (CoAP) [19] standards on web-accessible objects. The Findability Layer aims to solve the problem of “given an ecosystem of billions of smart things, how do we find their services to integrate them into composite applications?” [16], *i.e.*, to enable searching and finding relevant services and data. The focus of our paper is to survey the state-of-the-art research corresponding to this layer. The Sharing Layer focuses on privacy and security issues and the Composition Layer is concerned with composing applications based on discovered services, e.g., automated business process composition [20] and sensor data augmented with data aggregation [21]. For more detailed discussion on these two layers please refer to [16].

According to the WoT system model, the Findability Layer is the bridge between Web-accessible objects and their services to applications. This implies the significance of efficient and effective search technologies for WoT applications. Guinard *et al.* identify several challenges for providing search on WoT [16], for example, smart objects do not have many indexable properties (in contrast to textual information for searching documents); smart objects always contain contextual information, such as their geographical locations (coordinates), descriptive positions (e.g., Room B on Floor 1), or current owner; mobility of smart objects, such as movement of smartphones, will lead to continuously changing contextual information; fast-changing data is not suitable for scheduled indexing as in traditional search engines.

WoT applications have different objectives, purposes and scope, which can be translated into different requirements for search techniques. To provide the readers a better overview of the search techniques, we provide a mapping of the search requirements and techniques with the WoT domains and applications, as shown in Table 1. The classification of the applications is based on the W3C Web of Things Interest Group’s unofficial report [22]. In this article, we review, categorise and analyse the existing search techniques for different applications in the WoT and discuss how they address the requirements and challenges. Furthermore, we examine the recent trends and point out some of the future research directions.

## 3. Metrics for Evaluating Search Techniques

The WoT is a highly dynamic and evolving organism composed of billions of interconnected “Things”, consequently, the scope of the research in search techniques for the WoT is broad. Individual research work may just focus on a narrow aspect of this broad area, e.g., scalability, knowledge representation (to enable semantic search), stream handling, dynamicity *etc.* To gain a quick while in-depth overview of the research, it would be better to define some prominent metrics or dimensions against which the existing works can be compared. These metrics are explained as follows:

*Data format*: indicates representation of the data.
*Access approach*: refers to how clients of the search functionality can access the search results.
*Search type*: refers to the fundamental search techniques based on which the search systems are developed, such as keyword-based search, Structured Query Language (SQL)-like query, indexing, spatial search, or continuous query. The core techniques employed are important in determining the efficacy of the search.
*Scale of experiments*: indicates the scale of the experiments performed in a particular research work, e.g., the number of sensors and entities, or the amount of data, *etc.*, if the information is available.
*Dynamicity*: the term refers to the factors which have direct or indirect impact on the status, states or values generated by the “Things”, such as device registration, mobility (change of geographical locations), or sensor hardware fault [23,24]. It is used to indicate whether a search mechanism provides support to handle the problems caused by the highly dynamic WoT environment. In the WoT, the objects’ status and the values they produce may change rapidly. New incoming data may cause the mechanism to undergo extensive computations (leading to progressive performance degradation) or repeated changes in its underlying infrastructure (e.g., the index structures).
*Architecture*: points out whether a search platform is designed or the experiments are performed in a centralised or distributed manner (or both).
*Implementation*: indicates which programming languages/models are used to implement the search techniques or systems.

Other than the metrics mentioned above, some researchers also compare aggregation type and security support of search [15], query time, query accuracy, entity mobility and status, as well as targeted users [11]. For this paper, these characteristics are not considered as they do not apply to most of the reviewed techniques. 

Table 2, Table 3 and Table 4 provide a summary of the surveyed works against the metrics. As can be seen, various data formats have been used to model the WoT objects. Among them, many choose data formats that support semantics, which enables interoperability and automated reasoning, at the cost of some additional complexity. With respect to access method, REpresentational State Transfer (HTTP REST) and SPARQL Protocol And RDF Query Language (SPARQL) are the two most popular approaches due to their simplicity of implementation. The reviewed techniques employ many different search techniques, varying from keyword-based search to spatial search, semantic query, *etc.* More than half of the studied works aim at providing search functionalities on a global scale. It is interesting to see that many search techniques have taken dynamicity into consideration in their design, implying that the research community has been well aware of the distinctive charateristics of search on WoT.

Both centralised and distributed architectures (or combinations) are prevalent in the existing systems: a centralised architecture is efficient in process control at a single point whereas distributed architectures fit the characteristics of WoT best. Another notable finding is that most systems are implemented in Java, this can be largely attributed to the availability of many Java-based open source tools and APIs.

## 4. Classification Viewpoint—Basic Principles

Search as a topic has been investigated for decades; but existing techniques cannot be directly applied and need to be adapted to support search in the WoT. This section provides a classification that focuses on the underlying techniques and principles essential for search in WoT. Two broad categories of methods can be identified according to this classification, *i.e.*, indexing and clustering.

### 4.1. Indexing

Indexing is a technique that organises search key values and addresses of objects into catalogues to enable efficient lookup. The search functionality is provided by scanning the catalogue first and then locating the desired objects via the addresses in the catalogue. In the WoT, data objects are usually described according to a pre-defined knowledge representation model. Such descriptions contain useful textual information e.g., functionalities or geographical locations. Two main types of indexing approaches can be identified: text indexing and spatial indexing.

#### 4.1.1. Text Indexing

Text-based search techniques originate from the field of information retrieval. The overall search process can be roughly divided into two steps: indexing and searching. Indexing is used to scan all the words in documents (which can be done offline) to create an inverted index. Each term is linked to addresses pointing to the locations of the documents or the occurrences of the terms in the documents. During the search step, the index is first scanned; once the desired term is found in the list, the address of the documents can then be located. To reduce the size of the index and to improve the search efficiency, in practice, some specialised indexing data structures (such as the B-tree [82]) are used.

Data and objects in the WoT are usually annotated with textual descriptions according to some carefully designed knowledge representation models. The descriptions can include valuable information related to functionalities, environment, location, performance (with keywords such as “tolerant to noise” or “high resolution”), and other context information of the objects. These kind of textual descriptions can be crawled and indexed to build simple, text-based search systems. For example, Global Sensor Networks (GSN) [25], SenseWeb [29], OSIRIS [38], Dyser [30], Microsearch [31], Snoogle [32], and IoT-SVK [37] (which is a hybrid real-time search engine framework for the Internet of Things based on Spatial-Temporal, Value-based, and Keyword-based Conditions), all apply text based indexing and searching. Techniques for text indexing can also be used for indexing values, e.g., LiveWeb [33] and IoT-SVK [37] apply such indexing techniques for sensor values and provide search functionalities over the given values.

In addition, text-based indexing can also be combined with existing standards to provide search or discovery of smart objects over the Web. For example, the Domain Name System (DNS) [18] can be utilised and extended with indexing techniques for searching smart objects [34,35,36]. The HyperCat [83] server also manages and stores catalogues to offer search with simple criteria, lexicographic range search with sortable string format, *etc.* These approaches exploit the existing Internet infrastructure, hence support large-scale deployments. However, it is difficult to embed complex descriptions and rich semantics to the WoT objects through these standards, which may restrict the functionalities of WoT applications built on them.

Research initiatives such as GSN [25] provide APIs for users to publish sensors with descriptions. This approach offloads the annotation efforts for describing sensors to sensor publishers. However, since the descriptions are added by individual users manually, sometimes they tend to be inconsistent with each other and the accuracy cannot be guaranteed. Overall, text indexing is extremely easy to implement and can be used in most of the situations, but the most obvious limitation is the low search precision due to ambiguous descriptions.

#### 4.1.2. Spatial Indexing

WoT objects and data have a strong focus on locality; this implies that spatial information is vital for description and search. In some cases, spatial information and other features of objects and data are separated to provide search services [23,37,38,39,40,41,42]. Spatial information is typically represented by latitude and longitude coordinates (sometimes including altitude as well). The two-dimensional data cannot be effectively processed by text-based indexing structures as the latitude and longitude values tend to be different even for objects located near to each other.

Two or more dimensional data can be mapped to and indexed by one-dimensional keys by using space filling curves. Two such examples are GeoCENS [40] using peano curve to search for events and Zhou *et al.* work [42] using Geohash (Z-order curve) to search for O&M data and objects. The technique used in the latter work enables both historical and near real time search for data generated by both fixed and mobile objects.

Objects or data points can also be indexed by tree-like data structures based on spatial indexing techniques. For example, R-tree [84] is often used for indexing ranges. Approaches implementing R-tree based technique and its variants [23,37,38,39,41] index the Minimum Bounding Rectangles (MBR), which enclose the location range of all the children of a node. The nodes whose range intersects with the location in the query are retrieved. A known drawback of the R-tree based methods is the limited scalability; when a large number of locations of the physical entities are indexed, the MBR are likely to change frequently, which may negatively affect the indexing and query performance. The work in Wang *et al.* [23] alleviates this problem by indexing the gateways (in which semantic repositories are implemented to store the sensor descriptions) instead of individual sensors. Thus, changes of individual sensors (e.g., geographical properties) are constrained within the bounds of the gateway. This eliminates the necessity of frequent updates on the spatial indexing structures.

### 4.2. Clustering

Clustering-based search techniques first group the “things” or data into clusters, usually in an offline phase, and then execute queries in the selected cluster. Since the number of things or the amount of data in one cluster is relatively smaller, the overall query process is reasonably efficient. With location being a key property for objects and data in the WoT, geographical information or relative location plays a substantial role in clustering-based discovery methods.

#### 4.2.1. Location-Based Clustering

Recent research on IoT semantic modelling and knowledge engineering emphasises the importance of location [43] by defining semantic relations between IoT services and geographical locations (e.g., global location, local location and geographical coordinates). This modelling approach enables IoT service discovery based on the linked IoT data [44].

An indoor location-based (room, building, floor, *etc.*) search mechanism is proposed by [45] in which a hierarchy of semantic gateways is implemented. The gateways encapsulate semantic service descriptions of IoT objects within their geographic scope. Scalable search is provisioned through routing tables (constructed by recursive clustering of the semantic descriptions) which perform request matching and forwarding. However, this approach does not consider the cost of routing table maintenance (*i.e.*, update of service descriptions) due to the potential mobility of IoT objects. Mayer *et al.* [46] consider logical identifiers for places aiming to structure nodes in a hierarchy, with interactions restricted to direct communication between neighbourhood nodes to ensure scalability. The search process can be performed either at a local node or a distant node (based on query routing).

The geocasting-based approaches [47,48,49,85] provide capability of sending a query to nodes within a range of location in a distributed architecture. These approaches do not have concrete clusters thus are more flexible for mobile nodes, and are often used in Vehicular Networks [47,48]. However, the reliability of the query is hard to be guaranteed as available services cannot be known through geocasting techniques only. In addition, query response may need extra techniques to send messages to the query sender, especially mobile ones [47,48].

Location is one of three themes (the other two are thematic and temporal aspects) considered in [5] to associate IoT services to real-world physical entities. The authors propose a geographically distributed, federated architecture of cooperating nodes with local reasoning capabilities to manage the large number of IoT devices.

#### 4.2.2. Non-Location-Based Clustering

Location-based clusters can be inflexible on many occasions due to mobility of objects. Other than location-based clusters, Christophe *et al.* [50] cluster query requests into application invoked query and human accessed query. By specifying different requirements on the two categories, the system provides different services to the search functionality. In [51], the authors provide clustering of IoT services based on the Google Similarity Distance. In conjunction with a skewness-aware clustering tree for spatial query and a one-dimensional index for temporal feature, a compounded query for IoT services is enabled. However, the evaluation results of the clustering process show that the method does not scale well as the number of IoT services increases to certain values. Ebrahimi *et al.* [52] provide Sensor Semantic Overlay Networks (SSONs) for sensor search. SSONs cluster sensors based on their context information, which is defined based on the Semantic Sensor Network (SSN) Ontology. The authors propose an ant clustering algorithm, AntClust, for building clusters. User queries are sent to the most similar cluster for obtaining results. An adaptive strategy with changing threshold is used to maintain performance in the dynamic IoT environment and to control when to re-initiate the clustering process. As clustering techniques often require a time-consuming offline computing phase, they are more suitable for objects with relatively static structures, or within a specific scope. They are also vulnerable to dynamicity, e.g., a large amount of new incoming data due to changes in surrounding environments, which may lead to the clusters being frequently re-computed.

## 5. Classification Viewpoint—Data/Knowledge Representation

The Web of Things aims at making information about the objects and data generated by the objects accessible through Web standards. The Semantic Web aims at providing semantics and interoperability for any type of resource on the Web to build a Web of Data. The research communities have recognised the importance of Semantic Web techniques (e.g., knowledge representation formalism and automated reasoning to derive new knowledge) in realising intelligent services with connected objects in the IoT and WoT [44,53,54]. In this section, search techniques are explored from the perspective of data/knowledge representation, with a particular focus on the use of the Semantic Web technologies, *i.e.*, Linked Data and semantic streaming data.

### 5.1. Search and Query on Linked Data

Linked Data is an important concept for the vision of a Web of Data. Sir Tim Berners-Lee proposed the Linked Data principles, which suggest to publish data in standard formats (such as in Resource Description Framework (RDF) [86]) and to access it through existing Web standards (Hypertext Transfer Protocol (HTTP) look up and SPARQL query [87]). More importantly, the data items should be linked to each other and to other resources wherever applicable, to add semantics to the original data [88]. Linked Data offers the capability of linking data items with their Uniform Resource Identifiers (URIs), making it possible to build interlinked and distributed datasets. This subsection discusses centralised and federated (distributed) approaches to access the Linked Data in the WoT.

#### 5.1.1. Centralised Approaches

This class of methods publishes semantic descriptions of the objects in the WoT as linked data in a centralised fashion. For example, the work in [53,55,56] merges sensor description information into a centralised repository, and provides SPARQL endpoints for accessing the datasets. The semantic descriptions can be linked to other data sources that contain geographical information to enable simple spatial search, for example, all sensors near to the points of interest in a city. The limitation is that the spatial search functionality can only support keyword-based search through filters in the SPARQL query at coarse levels.

As locations become increasingly important for objects and data in the WoT [89], some researchers extend the SPARQL language with spatial search capabilities [57,59,60]. These works either add a spatial ontology (such as World Geodetic System 1984 (WGS84) ontology [90]) or spatial properties (geom:geometry [57]) into the original ontology. They then use built-in functions of the triple store (such as OpenLink’s Virtuoso [91]) or build external functions to enable spatial search at finer levels, such as ‘within a region’. The search process can directly run SPARQL queries on the endpoints to get objects satisfying semantic restrictions as well as spatial constraints. Spatial index structures, such as R-tree, can be introduced to make the search even more efficient. A comparison of different semantic Web tools for spatial query is provided in [92].

Centralised approaches enable fast response to queries, however, they require the data to be stored in a centralised repository. This introduces a number of limitations, for example, single point of failure, duplication of data (which introduces difficulties in maintaining the status of all objects and data), and poor scalability.

#### 5.1.2. Federated Approaches

One benefit of Linked Data is that it allows the data storage to be distributed over the Web while maintaining semantic links between the resources. Obviously, centralised approaches do not take full advantage of Linked Data. One can utilise federated query techniques to enable transparent query over distributed repositories.

In federated search, the original query is decomposed into a number of sub-queries, and the search system helps determine which source or dataset can provide potential answers for a sub-query. The sub-queries are sent to the relevant repositories to retrieve intermediate results, which are finally federated to compose the final results. Processing of the sub-queries is unlikely to be efficient without a proper execution plan. Existing implementations [61,62,63,64,65] provide different query optimisation techniques for the query process, for example, optimisation based on complete knowledge and statistics of datasets (DARQ [61], ANAPSID [62], SPLENDID [63]), or heuristic ([65], FedX [64]).

It is worth noting that most of the above implementations are based on SPARQL 1.0. The newer version, SPARQL 1.1 [66] supports federated query capability by expressing queries across diverse datasets, and has been approved as a W3C recommendation (for more information about the syntax of SPARQL 1.1, please refer to [67]). In addition to the previous query constructs, it uses the *SERVICE* statement to declare a remote SPARQL endpoint. The work in ANAPSID [62] extends SPARQL 1.1 by providing the *agjoin* and *adjoin* operators to further reduce execution time, enabling a query to be answered even when a source is blocked.

Federated search addresses some of the limitations of the centralised search. The challenge is how to design appropriate query optimisation techniques to enable efficient distributed queries. Furthermore, its performance is subject to transmission delays and network bandwidth. Related challenges and future directions are discussed in [93], for example, metadata management, caching results, and adaptive query processing in federated search.

### 5.2. Search over Streaming Data

WoT comprises of both static and dynamic data, for example, metadata describing sensors or objects is relatively static and changes infrequently; while data generated by sensors and objects are dynamic and of streaming nature. Streaming data has its own characteristics, for example, the speed at which data is generated may change (because of change of the application requirements), or the data flow may stop and recover occasionally (recurrence). In some delay-tolerant networking applications, data might not arrive in order, or arrive later in bursts. Therefore, it requires specialised techniques that can quickly process the continuously generated data for indexing and querying. Moreover, the data values are always tagged with time stamps and (often with) spatial information as well. This subsection presents the recent works on searching over streaming data through relational database mapping and semantic modelling.

#### 5.2.1. Relational Database Mapping Approaches

Data Stream Management Systems (DSMS) aim to provide functionalities for managing streaming data based on traditional relational database systems. In the context of WoT, the research mainly focuses on providing languages and facilities to support continuous queries [25,68].

The GSN framework [25] is a DSMS that offers ad-hoc data access APIs to virtual sensors stored in the GSN system. The queries allow sliding windows through explicitly defined temporal parameters. SenseWeb [29] supports queries for sensor data streams based on type, location and descriptions of sensors. Ontology-based query of live sensor data is presented in [68]. The authors use the R2RML language (RDB-to-RDF mapping language) for mapping streams stored in relational databases to ontological schemas. The virtual RDF streams can be queried using an extended SPARQL language that supports time windows. The authors also validate their method by using query mapping to retrieve data from existing DSMSs such as ESPER [94], GSN and Xively [95].

While these works provide a useful approach for ontology-based query of sensor O&M time-series data, the actual query processing and O&M data collection/storage is delegated to the DSMS. The functionalities offered by this class of methods are generally limited to those provided by the underlying relational databases.

#### 5.2.2. Semantic Modelling Approaches

An alternative approach is presented in [44], where semantically annotated sensor O&M data is transformed to streams and is assigned a unique identifier. The naming mechanism is based on the location, quality and start time of the measurement. To deal with the large amount of annotated data, a K-means clustering algorithm is applied to distribute the data among different repositories. Resolution is done by finding the nearest cluster to identify the repositories that are likely to contain the data sought by the queries. However, queries requiring time window and data aggregation functions are not supported.

As a lot of streaming data has been published as RDF data [44], efforts to extend the SPARQL language to answer continuous queries for streaming data have been undertaken. Some of the examples include C-SPARQL [69], EP-SPARQL [70], CQELS [71] and SPARQLstream [68]. Among them, SPARQLstream and C-SPARQL store data in DSMS, and provide translation services to enable continuous queries. In contrast, CQELS uses its own native processing model in the query engine, which can dynamically adapt to changes in the input data. Linked Sensor Middleware (LSM) [27] is an application based on CQELS. LSM is also integrated with eXtended Global Sensor Networks (XGSN) [26] in OpenIoT [28].

Semantic modelling based methods add rich semantics to the WoT data and provide more powerful reasoning capabilities than relational databases. However, the data model is relatively more complex and requires extra computation during runtime. To alleviate the problem, Shin *et al.* [72] propose query adaptive techniques to filter out semantic streaming data that is not related to registered query to reduce both the size of storage and the query response time.

## 6. Classification Viewpoint—Contents Being Searched

This section provides a review to the search techniques for WoT from the content perspective as different content types entail different search methods. Due to the importance of sensors and sensor networks to WoT and IoT, this section focuses on the following three types which form the basic contents of the WoT: information about sensors (*i.e.*, the descriptive and contextual information for sensors), Observation and Measurement data (*i.e.*, data produced by different kinds of sensors), and information about entities (information objects in the WoT, e.g., city, point of interest, patient, or smart home). From an object-oriented or semantic modelling view, a sensor is also a kind of entity in the WoT; however, the discussion in this section distinguishes sensors from entities due to their particular importance.

### 6.1. Search for Observation and Measurement Data

O&M search aims to find the desired measurement data based on pre-defined requirements, for example, within a specific time range in a particular location. One approach is to perform a sensor search first and then retrieve the O&M data from selected sensors. However, it may not be effective for mobile sensors, whose spatial values might change frequently over time. Another approach is to perform direct search in streaming databases which store large amounts of O&M data collected from sensors. The requirements for real-time streaming data and historical data are considered separately in this subsection. Search techniques for streaming data have also been discussed in Section 5.2. Most of them can support short-term historical data queries (bounded by the given time windows), but normally not long-term historical data.

#### 6.1.1. Instantaneous Data

Real-time data is important for many applications; for instance, users may need to know the availability of meeting rooms or the nearest car parks with available parking space. A lot of research has been undertaken in designing and developing real-time O&M data search. The work in SensorMap [39] allows users to first search sensors on a location map by specifying the sensor type. Once the matching sensors are found, the latest values generated by those sensors are retrieved. Liveweb [33] creates an index on real-time data in a tree structure to enable efficient search. GeoCENS [40] applies space filling curve on the spatial information of sensors, and implements a hybrid Peer-to-Peer (P2P) architecture to support spatial search for sensors. The O&M data is then obtained from the selected sensors. IoT-SVK [37] integrates and symbolises sampling values into keywords, then applies Value-Symbolized Keyword B^+^-tree to support O&M value queries. However, due to transmission delays or communication failure, real time data services sometimes cannot guarantee data freshness, especially in time-critical applications. The work in [71] combines search techniques with a short-term prediction algorithm to mitigate this problem.

#### 6.1.2. Historical Data

Historical data may not seem important in day-to-day lives, but they are vital for data analytics and predictive modelling. The work in LSM [27] provides facilities and services for storage and query of historical data. These search services generally assume that the context of O&M data is static and not likely to change frequently. IoT-SVK [37] indexes both O&M values and time stamps to support historical data search.

Spatial information is one of the most important context information crucial to the retrieval of O&M data. As sensors and smart devices are increasingly attached to mobile objects (buses, humans, *etc.*), spatial information of O&M data may change frequently. Traditional methods cannot provide effective search services for O&M data generated by mobile objects. To address this issue, Zhou *et al.* [42] provide a data-centric framework which encodes spatial features into the O&M data and stores it into a cloud based time-series database. The framework enables search for historical and near real-time data collected from both static and mobile sensing sources. A potential challenge is the storage of O&M data as it is continuously generated from an increasingly large number of static and mobile objects. One future research direction is the design of more effective information abstraction methods for real-time streaming data, which largely preserve the valuable information contained in the original data and at the same time reduce the needs for large storage space. The recent research on NoSQL databases and cloud-based storage can also be used to alleviate the problem.

### 6.2. Search for Sensor Information

In many applications, especially those that need continuous data, it is computationally expensive and time-consuming to search for O&M data directly and repeatedly. A much more efficient approach is to identify the best sources which can provide the needed data and to subscribe to them. The objective of sensor search is to find the right sources which can provide the O&M data (preferably high quality, or with less computation cost) needed in an application. The existing methods can be categorised into two groups: context-based (e.g., search based on locations and types of sensors); or content-based, (e.g., search sensors that generate certain values).

#### 6.2.1. Context-Based Sensor Search

Context-based sensor search relies on various type of contextual information available in the descriptions of sensors and services. Microsearch [31] and Snoogle [32] offer search services for sensor nodes by indexing the sensor descriptions. Jirka *et al.* [38] provide sensor search based on keywords as well as indexed geographical locations. The works in [53,56] publish sensor data (sensor descriptions and sensed data) following the Linked Data principles. The published data is also linked to concepts in existing datasets, such as GeoNames [96], to extend the original descriptions with geographical information. This allows users to discover sensors based on named locations.

Other than location and type, context may include information related to quality of service of sensors, such as accuracy, reliability, delay, *etc.* CASSARAM [73] proposes search and selection of sensors based on user expectations and priorities over quality of service related information. Sensors are modelled with contextual properties based on the Semantic Sensor Network Ontology (SSN Ontology) [97]. A weighted Euclidean distance based indexing technique is used to measure how similar the description of a sensor is to the user requirements. The authors also apply an optimised parallel processing method to enable distributed search over different server nodes to acquire highly ranked sensors. Shah *et al.* [74] provide a search service based on multiple features in a P2P network, which consist of distance, energy level, communication cost, computation cost, *etc.* Selection of the sensors is based on the Euclidian distance of weighted features of sensors and the user requirements.

Sensor search techniques based on context information are effective in retrieving a list of potentially useful sensors. However, the limitation lies in the availability of the contextual information. For example, while geographical information can be obtained in straightforward ways, quality of service related information is often difficult to capture for individual sensors. Furthermore, carefully designed ranking algorithms are needed to select the best ones from all the retrieved sensors.

#### 6.2.2. Content-Based Sensor Search

Content-based search aims to find sensors based on the values generated by sensors. The work by Elahi *et al.* [77] leverages human periodic behaviour and predicts the sensor output at future points. The results are then used to estimate the probability that a sensor matches the query requirements. The work by Truong *et al.* [76] calculate the similarity scores between sensors and a given sensor (used as a query) with the output of sensors. The matched sensors are further ranked based on the fuzzy set theory. Although research in this line is not as popular as context-based one, it represents a useful approach in some applications, for instance, monitoring whether sensors are functioning properly, identifying sensors that need maintenance, deploying new sensors, or defining new services based on particular sensors.

### 6.3. Search for Entity Information

Entity search has potential usage in a wide range of human-centric applications whose primary tasks are to facilitate interactions between human users and intelligent systems. As sensor is a particular type of entity or object, it is not surprising that techniques used for searching entities are similar to those designed for sensor search in many ways. In almost all applications, sensors and various kinds of entities (e.g., a parking lot, smart office, or a patient) are always associated to each other. The work in Dyser [30] and SPITFIRE [75] in fact provides both sensor search and entity search. The existing research on searching entities is also classified into context-based and content-based.

#### 6.3.1. Context-Based Entity Search

Knowledge representation plays important roles in searching both sensors and entities. In sensor search, contextual information about sensors can be represented by using the widely used models, such as the SSN ontology [97], or Sensor Model Language (SensorML) [13]; however, currently, there is no standard knowledge representation models for entities, which might introduce some interoperability problems in the search systems in the WoT.

DiscoWoT [81] is a general semantic discovery service for Web-enabled things. The descriptions of the things can be created by arbitrary users through a given Web-based interface according to its internal description structures, which contain the basic, contextual and product-related information about the resources. Qian *et al.* [79] provide an IoT Search Engine to enable search for Radio-Frequency IDentification (RFID) objects in real-time. The engine consists of several modules: an index, update module, query module, and security module. The index module implements a distributed indexing and storage component. The update module can handle different update operations (*i.e.*, add, delete, and modify). The query module provides an interface to handle query requests and representation of results. The security module applies the Elliptic Curve Cryptography-based algorithm [98] for encryption and decryption, providing authentication and protection of vital entities. Gander [78] is a middleware for pervasive computing environment that is able to capture the context of data items and to provide real-time search capability for nearby entities. It applies sampling on the spatiotemporal information through peer-to-peer methods. The context used for search includes relationships among data items and their surrounding environments.

Compared to sensors, entities significantly vary in size, scope, type and capabilities. It is difficult to design a comprehensive knowledge representation model for all kinds of entities in the WoT. A general model (e.g., an upper-level ontology) is not able to capture more specific knowledge of the various entities in different domains. Therefore, applications implementing entity search are usually limited in terms of domain and scope (e.g., specificity).

#### 6.3.2. Content-Based Entity Search

Content-based entity search can find entities based on their states or status. Different from content-based sensor search which directly searches through raw O&M data, content-based entity search requires entities’ states or status to be derived from the O&M data first.

Dyser [30] supports searching real-world entities that are in some specific states, e.g., available parking slot. In Dyser, an index is first created based on the metadata of sensors and entities. During the query process, a number of sensors relevant to the entity of interest are retrieved first, and then the state of the entity is derived based on the values collected from its associated sensors. The matched entities are sorted by the ranking technique proposed in the authors’ previous work [77]. The search process stops when enough (top k) matched entities are found. The prediction model used in Dyser periodically computes the states of entities based on the measurement data from sensors. The work in Mietz *et al.* [80] applies Bayesian Network to automatically infer the states of the entities. SPITFIRE [75] infers states of things from the embedded sensors based on their semantic descriptions. One notable feature of this work is that it employs a short-term prediction model. Because of the dynamicity of the WoT, the status of the returned entities may even change due to transmission delays. The short-term prediction can resolve this issue effectively.

## 7. Discussion

This section presents lessons learnt from the comparison of the existing work and the experiences gained from the authors’ involvement in some of the large EU research projects on WoT and IoT. The discussion is provided following the classification of the existing search techniques.

### 7.1. The Basic Principles Perspective

Traditional spatial indexing techniques are good at indexing static objects; however, sensors and smart objects are increasingly becoming mobile, e.g., smart phones, buses equipped with sensors, *etc.* These mobile sensors lead to opportunistic sensing, which provide sensing data in extended areas. However, very few spatial search mechanisms consider mobile objects, e.g., trajectory search [99,100]. As mobile sensors and opportunistic sensing become more and more prevalent, search methods for the WoT applications need to pay special attention to them.

Searching based on existing standards is a good practice in designing search systems in WoT and shows a promising direction, for example, the DNS-based approaches can exploit the current Internet infrastructure, in which Web crawlers can be designed to collect descriptions of IoT devices. Existing Web standards are used mainly for resources and devices discovery, which is based on proposed or existing registries deployed on the Web. These registries manage identifiers and metadata of sensors, entities, and their services, offering simple search functionalities (such as keyword match) within the scope of registries. Distributed search on a large scale relies on peer-to-peer overlays covering these registries. More complex search services may require additional search techniques, such as Elasticsearch [101], to be integrated.

Due to the dynamicity (e.g., changing physical environment factors, mobility, device failure, and opportunistic sensing scenarios) and open tasks (local area data analytics, personalised smart services, *etc.*), search in the WoT should be implemented along multiple dimensions, *i.e.*, temporal, spatial, and thematic. This is one of the most significant differences to other traditional search applications. The work in [37] follows this principle by combining different indexing techniques and designing a search method based on time, locations and values, and reports encouraging results. It shows that developing search methods exploiting the temporal, spatial and thematic properties of the WoT objects and data simultaneously is a promising direction.

Most of the reviewed works implement middlewares based on the basic search principles for easy deployment, such as OpenIoT [28], GSN [25], GeoCENS [40], Geospatial Indexing [23]. The middleware platforms hide the comlexity and heterogeneity of the representation of and communication among objects and sensors, and offer easy-to-use search functionality based on stardard service interfaces. However, evaluation and comparison of the existing methods are difficult. We have done a lot of investigation on the possibility of performing such evaluation and comparison among all surveyed works: we checked if the systems in the reviewed papers are still accessible through the provided Uniform Resource Locators (URLs) or SPARQL endpoints and have clear input specification (so we could perform some experiments to measure the throughput and scalability). Unfortunately, we only got a very small number, which makes the comparison and evaluation insignificant. Another difficulty is that most of the works are performed in a closed environment and the datasets are not publicly available. Moreover, the reviewed works implement different methods and use different data formats and datasets.

### 7.2. The Data/Knowledge Representation Perspective

Although this study differentiates the centralised and distributed methods, the line between them diminishes in real systems. Based on the review of the related work, it is straightforward to see that a combination of both methods has clear advantages. A good practice is to maintain metadata on WoT objects and data in a relatively more centralised repository and to store the more dynamic, streaming data in distributed storages. The metadata can be published as linked data, which links to other data items in different sources (e.g., domain knowledge bases and existing linked data cloud).

With respect to evaluation strategies, centralised, federated, and streaming based approaches need to be considered differently. Even methods falling into the same category might be difficult to compare, for example, most centralised approaches extend the Linked Data query with geospatial functionalities, e.g., LinkedGeoData [57], OWLIM-SE [59], GeoSPARQL [60]. However, the internal knowledge representations of these research works are different and cannot be directly compared without translating one representation to another. Federated approaches evaluate queries on distributed datasets (DARQ [61], ANAPSID [62], SPLENDID [63], FedX [64], Federated Query Implementation [65]). Fedbench [102] provides a benchmark test for response time in federated approaches. A comparison study on response time is provided in SPLENDID against DARQ and FedX. It is reported that in that particular experiment, FedX outperforms others in most queries and SPLENDID performs competitively. Streaming based approaches are concerned with continuous analysis over sensor streams (e.g., C-SPARQL [69], EP-SPARQL [70], CQELS [71]). CQELS provides a benchmark for continuous query and compares the results against C-SPARQL and ETALIS (EP-SPARQL). In [71], it is reported that CQELS outperforms the others and its performance is stable during the experiment.

As the size of the streaming data is likely to be much larger than the size of the metadata of WoT objects, efficient search remains a challenge. The work in CQELS [71] presents a promising approach for searching semantic streaming data, which is independent of the DSMS and has the potential to make use of the full capacity of semantic representation and reasoning. However, in the vision of the “Big Data”, the current efforts are far not enough. One of the trends is to leverage the recent development in big data processing platforms in designing search techniques for the big streaming data of WoT.

### 7.3. The Content Perspective

From the review of the existing research, one can see that search techniques for Observation & Measurement data, sensors and entities tend to converge, especially for some WoT applications that need different ways to access data generated about the physical world. However, as the contents being searched are significantly different, we are not able to find any comprehensive evaluation for the methods under this category, which remains as a future research topic. The review also highlights the importance of the semantic models (in particular, the lightweight models) in knowledge representation. Besides the ontologies designed for sensors, the IoT Domain ontology provided in IoT-A Reference Model [103] is becoming more and more popular and finds its application in diverse areas such as business process modelling [104], dynamic association derivation between ICT and real-world objects [5,105], service discovery [41,106], service selection and ranking [107] and test case derivation for IoT service lifecycle management [108].

Current research on derivation of entity status mostly employs simple methods (e.g., to detect if a room is hot/cold for humans). The conclusion drawn from the sensor measurement data sometimes may be too coarse as the prediction methods do not take the differences of individual users or applications into consideration. The future research in entity and sensor search needs to look into the personalisation problem, which entails more sophisticated machine learning based methods. In particular, the research needs to pay more attention to the accuracy of the prediction, especially for time-critical applications.

## 8. Outlook

The study presented in the previous sections shows that many techniques from different research fields, e.g., information retrieval, semantic Web, database, and knowledge management, have been applied to the development of search methods in WoT applications. The major differences between “search in the WoT” and “search on the Web” are well understood in most of the research works. The research community has also recognised the complex nature of the search problems in WoT, for instance, dynamicity of the things and data (while documents on the Web are relatively much more static); the uncertainty (while status of documents is relatively much more stable); spatial and temporal properties (while these are not the primary concern of Web documents). To address these problems, researchers have applied different techniques, such as semantic modelling and description, spatial indexing, federated and peer-to-peer search. However, these efforts are far from being sufficient, especially with the pressure of challenges of the overwhelming amount of data produced by the sensors and smart objects in the WoT. Obviously, the data produced on the WoT is a kind of big data. To this end, the following future research directions can be identified:

*Big Data Search*: the term is used to indicate the need for designing and developing more effective and efficient techniques for searching the big WoT data. Although the existing research has built solid frameworks for search services, it has not seriously taken into account the potential problems introduced by the volume, velocity and variety of the big data, which are compounded by the dynamicity of the WoT. For example, the MapReduce programming model, which is fundamental to the existing big data processing platforms such as Hadoop [109] and SPARK [110], requires that a computation task should be expressed as computing sums of functions over the whole dataset. This often involves a lot of repeated and frequent inter-machine communications. The input to the sum functions are a large number of key-value pairs in which the keys need to have fixed representations. However, this is not practical and extremely inefficient for applications in the WoT with high degree of dynamicity, e.g., the spatial and temporal properties and associated objects of a sensing device may change frequently, making it difficult or impossible to compute a key for the O&M data from that sensing device. To make the big WoT search more efficient, some of the existing underlying building blocks in searching the WoT infrastructure might need to be redesigned by leveraging the innovative technologies developed in the big data research. The complexity of searching big data lies in not only the design of search methods, but also the methods for distributed storage, abstraction, processing and analytics. This direction interleaves with other research directions, which are explained in the following.

*Distributed Intelligence*: it is not possible to collect all the information available on the WoT, build a centralised index and provide the search functionalities. This is fundamentally different to the development of Web search engines, in which most of the Web documents can be crawled and processed in a powerful, centralised data centre. The concept of distributed intelligence in this context implies a framework in which search functionalities are implemented on distributed, autonomous units that can cooperate to provide transparent search services to end users or applications. The most notable advantage is that each individual distributed unit applies intelligent processing mechanisms locally to hide the effects introduced by uncertain and dynamic factors pervasive in WoT. Another advantage is that after the distributed processing, quality of the data can be guaranteed (e.g., missing values can be estimated through regression analysis, data items can be ranked based on the local criteria, or inaccurate values can be eliminated) and volume of the data can be significantly reduced.

*Information Abstraction*: Besides providing search for entities and O&M data, future WoT search techniques may also support the search for abstract data objects, which encodes richer information in compact representation. Such abstraction can be implemented on different levels of a search system, for example, data from different sources can be aggregated based on the spatial or temporal dimensions to create high quality data series; missing values can be approximated by data collected from similar sources nearby. With proper inference techniques, information abstraction techniques can help extract patterns or events, which are useful for many data mining and machine learning algorithms, as well as for developing knowledge oriented search (*i.e.*, semantic search) functionalities.

*Personalisation*: many WoT applications, such as smart cities and smart home, are in line with the concept of human-centric computing. The ultimate objective of such applications is to provide intelligent assistance to human beings to make their life quality better. However, the current research is mainly intelligence-centric and pays less attention to the differences between each individual human user. Geographical information has been used to develop some sort of personalised, location-based search services in some of the existing research works (e.g., to constrain the search scope by searching restaurants within 500 m to the user’s location). However, more personalised search mechanisms need to be developed by exploiting not only the spatial information, but also temporal information and individual’s preferences. Furthermore, personalisation also allows automated adaptation of the search results without requiring users to explicitly specify the search criteria.

## 9. Conclusions

The complex nature of the WoT entails specialised search techniques for not only the physical or virtual “Things”, but also the data produced by those things. During the past few years, many techniques have been developed, covering a multitude of functionalities and dimensionalities. In this survey, a taxonomy for these existing techniques is defined, which allows the readers to gain a better picture of the research landscape. The state-of-the-art is reviewed and the existing techniques are compared from different viewpoints. The paper does not compare the search techniques through benchmark evaluations, due to the unavailability of benchmarks that can handle all kinds of search techniques included in this survey. The review is followed by a critical discussion on the current limitations, best practices as well as some of the lessons learned. The paper also proposes several promising future research directions under the emergent challenges of big data on the WoT. The study will benefit the research community in understanding the state-of-the-art on search techniques in WoT and gaining insights into the future research challenges and directions.

## Figures and Tables

**Table 1:** WoT Applications and Enabling Search Techniques.

Domain	Applications	Search Requirements	Search Techniques
Smart Cities	Community-based Flood Monitoring; Mobile Applications; ollution Monitoring; Public Rental Bicycle System; Soil Monitoring; Water Level Monitoring; Weather Monitoring	Require search with multiple functionalities (e.g., search by descriptions, by locations, or others); Require managing fast changing and updating of sensing data; May require both real-time and historical data access; Require data analysis/prediction; Require handling of mobile objects and their states	Real-time Data Retrieval; Entity Search; Location-based Search; Context-based Sensor Search
Home Automation	Adaptive Building Smart Control of Washing Machine	Dynamical selection of the surrounding/affecting resources Discovery of devices	Entity Search Sensor Search
Manufacturing	Lifecycle Management for Industrial Automation Systems	The discovery needs to be enabled in a dynamic environment where physical resources appear and disappear during lifecycle phases.	Context-based Search
Smart Grids	Virtual Power Plants	Require dynamically finding operateable distributed energy resources	Entity Search
Network Management	Device, Network, and Application Management	Require finding all devices that have a certain set of properties.	Context-based Search

**Table 2:** Comparison of Search Techniques According to Basic Principles Classification (Abbreviations are listed in the footer).

Classification	Search Technique	Data Format	Access Approach	Search Type	Scale of Experiments	D^1^	Ar^2^	Implementation	Remarks
Text Indexing	GSN [25]	Virtual sensors in XML	HTTP REST	Keyword-based search, SQL query with time window	-	Yes	Cen	Java/MySQL	Extended as XGSN [26] for distributed data acquisition and semantic annotation. Integrated with Link Sensor Middleware (LSM) [27] in OpenIoT [28] project
Text Indexing	SenseWeb [29]	Sensor ontology (an extension of the namespace defined by OGC SWE)	Map display with text description	Keyword/spatial search	-	Yes	Cen	-	Many applications described but none accessible
Text Indexing	Dyser [30]	Virtual sensors (microformat)	URL/HTTP/HTML	Keyword-based/value-based	385 sensors over 5 months	Yes	Cen	Java/PHP	Query of state of real world objects; Computation required for state awareness, the scale is limited to a small number of sensors
Text Indexing	Microsearch [31] Snoogle [32]	Direct communication with sensor motes with built-in metadata	Virtual sensors in built-in format	Keyword-based top-k search	-	Yes	Dis	TelosB motes	Early search implementations with limited functionalities and limited scalability
Text Indexing	LiveWeb [33]	Sensor data in XML	HTML/AJAX	Real-time content search based on keywords, category, and value range	-	Yes	Cen	PHP/Java/Apache/MySQL	Practical implementation without evaluation Should fit in global scale
Text Indexing	lmDNS-SD [34]	Extended DNS record for sensors and objects	List of sensors	Resource directory based on DNS-SD	-	Yes	P2P	-	Existing Internet standards utilised, thus fit in Web scale
Text Indexing	DNS Search [35]	Extended DNS record for sensors	HTTP REST/WADL	URL and DNS-based (type/location)	250,000 zones	Yes	Dis	BIND/MySQL	Utilise DNS structure; fit in Global scale
Text Indexing Spatial Indexing	Mobile Digcovery [36]	Integration of Multi-types	JSON-based response	Multi-functional JSON-based query	-	Yes	Dis	ElasticSearch	Combination of search engine and Web Infrastructure
Text Indexing Spatial Indexing	IoT-SVKSearch [37]	Sampling values with metadata in Raw Data Store	Data in built-in format	Keyword-based (B^+^-tree), spatial-temporal (R-tree), value-based (B^+^-tree) search	140,800~352,000 sensors	Yes	Dec	PostgreSQL/file system	Multiple search functionalities supported
Text Indexing Spatial Indexing	OSIRIS [38]	OGC SWE Sensor model and data model	SWE services	Spatial, temporal and keyword-based search	-	Yes	Cen	JSI/Apache Lucene	Applications in Smart City domain Website of the project no longer accessible
Spatial Indexing	SensorMap [39]	Direct communication with web accessible sensors	Web services APIs	Spatial search (based on COLR-tree/crawling)	-	-	Cen	MSRSense toolkit	SenseWeb-based application, not accessible currently
Spatial Indexing	GeoCENS [40]	OGC SOS, SensorML, OGC O&M	SWE services	Spatial search (Peano space filling curves)	~40,000 sensors/procedures	Yes	P2P	-	Designed for sensor data from smaller organizations or individuals
Spatial Indexing	Geographic Service Discovery [41]	Services with geo-information	HTTP REST	Spatial search (R-tree index/category server)	3200 services	Yes	Dis	Java	R-tree is combined with distributed architecture
Spatial Indexing	FUTS Data Query [42]	Virtual Object ontology in OWL-DL	SenML/JSON	Spatial search with time window/sensor type	20,000 sensors	Yes	Cen	Java	Cloud database for huge data storage
Spatial Indexing	Geospatial Indexing [23]	OWL-S	SPARQL endpoint	Semantic query (R-tree index)	10,000 services	Yes	Dis	Java	R-tree and Semantic query combined
Location-based Clustering	IoT Platform [43]	SSN ontology/OWL-DL	SPARQL endpoint	SPARQL query	-	Yes	Cen	Java/Apache Tomcat	Demo in smart building, could be applied to a larger scale as well
Location-based Clustering	Linked Sensor Streams [44]	RDF stream data	SPARQL query response	Type/location based query (based on clustering)	20,000 stream data	Yes	Dis	Java	Evaluation provided for clustering, not for search
Location-based Clustering	IoT Service Search [45]	OWL-S	Service access standard	Semantic-based query (based on clustering)	7500 services	No	Dis	Java Web Application/ Apache Tomcat	Mobile services may crash this architecture
Location-based Clustering	Web-based Infrastructure [46]	Microdata Microformats	HTTP REST	Keyword matching with scope	600 simulated sensors 100,000 resources	No	Dis	ApacheBench	Demo in smart building, mobile services may crash this architecture
Location-based Clustering	Geocasting [47,48,49]	-	-	Geolocation-based query	-	Yes	P2P	-	Flexible approach, no reliance on any model or architecture
Non-location-based Clustering	WoT Search [50]	Virtual Object ontology in OWL-DL	-	Search with user preference and geo-location (search by application or human)	-	-	-	-	Technique not implemented
Non-location-based Clustering	IoT Serv ices Indexing [51]	Web services (DPWS)	REST/SOAP	Functionality clusters, spatial query (SWC-tree), temporal query	1600 IoT-WS 50 clusters	Yes	Cen	C#/Matlab	Clustering process does not scale when IoT-WSs increase
Non-location-based Clustering	AntClust [52]	SSN Ontology	SPARQL query response	Ant clustering based on context	100,000 sensors	Yes	Cen	-	Good performance on query time but may need a lot of time to deal with incoming data, thus may not be suitable for real-time search

^1^ D is short for Dynamicity. ^2^ Ar is short for Architecture. Other abbreviations include AJAX: Asynchronous JavaScript and XML, Cen: Centralised, Dec: Decentralised, Dis: Distributed, DNS-SD: DNS Service Directory, DPWS: Devices Profile for Web Services, FUTS: Frequently Updated Timestamped and Structured, HTML: HyperText Markup Language, JSI: Java Spatial Index, JSON: JavaScript Object Notation, O&M-S: Semantically annotated O&M, OGC: Open Geospatial Consortium, OWL: Web Ontology Language, OWL-DL: OWL- Description Logics, OWL-S: Semantic Markup for Web Services, RDB: Relational Database, RDFS: RDF Schema, SenML: Sensor Markup Language, SML-S: Semantically annotated SensorML, SOAP: Simple Object Access Protocol, SOS: Sensor Observation Service, SWE: Sensor Web Enablement, WADL: Web Application Description Language, XML: eXtensible Markup Language.

**Table 3:** Comparison of Search Techniques According to Data/Knowledge Representation (Abbreviations are listed in the footer of Table 2).

Classification	Search Technique	Data Format	Access Approach	Search Type	Scale of Experiments	D^1^	Ar^2^	Implementation	Remarks
Centralised	Linked Sensor Data [53]	RDF	HTML/XML	SPARQL query	20,000 weather stations	Yes	Cen	Virtuoso RDF	Combination of Linked Data, sensors and sensor data
Centralised	IoT-DS [54]	RDF	SPARQL query response	SPARQL query	100,000 instances	Yes	Cen	-	Proposed IoT directory is similar to IoT Gateway
Centralised	SemSOS [55]	RDF	SML-S/O&M-S	SOS query mapping to SPARQL query	20,000 sensors	No	Cen	Apache Jena	Mobile sensors are ignored
Centralised	SemSOS [56]	SWE based model in Linked Data	OGC SWE SOS	Named location based query	20,000 weather stations	No	Cen	RDF2Go/Sesame	Different implementation of SemSOS
Centralised	LinkedGeoData [57]	RDF	SPARQL endpoint HTTP REST	SPARQL-like query	-	Yes	Cen	Java	Enables geolocation query as part of a SPARQL query Online version available at [58]
Centralised	OWLIM-SE [59]	RDF/RDFS/OWL	SPARQL endpoint	SPARQL-like query	-	Yes	Cen	Java	Enable geolocation query as part of a SPARQL query
Centralised	GeoSPARQL [60]	RDF	SPARQL endpoint	SPARQL-like query	-	Yes	Cen	Apache Jena	Enable geolocation query as part of a SPARQL query
Federated	DARQ [61]	RDF datasets	SPARQL query response	SPARQL query	-	No	Dec	Java	Requires service descriptions of datasets Supports limited queries
Federated	ANAPSID [62]	RDF datasets	SPARQL query response	SPARQL query	-	No	Dec	Python	Requires predicates of datasets
Federated	SPLENDID [63]	RDF datasets	SPARQL query response	SPARQL query	-	No	Dec	Java	Requires VoID of datasets
Federated	FedX [64]	RDF datasets	SPARQL query response	SPARQL query	-	Yes	Dec	Java	Easy to be extended as only the endpoint is required
Federated	Federated Query Implementation [65]	RDF datasets	SPARQL query response	SPARQL query	-	Yes	Dec	Java	Easy to be extended as only the endpoint is required
Federated	SPARQL 1.1 Federated Query [66,67]	RDF datasets	SPARQL query response	SPARQL query	-	Yes	Dis	-	W3C Recommendation for Federated Query
RDB Mapping	GSN [25]	Virtual sensors in XML	HTTP REST	Keyword-based search, SQL query with time window	-	Yes	Cen	Java/MySQL	Extended as XGSN [26] for distributed data acquisition and semantic annotation. Integrated with Link Sensor Middleware (LSM) [27] in OpenIoT [28] project
RDB Mapping	SenseWeb [29]	Sensor ontology (an extension of the namespace defined by OGC SWE)	Map display with text description	Keyword/spatial search	-	Yes	Cen	-	Many applications described but none is accessible
RDB Mapping Semantic Modelling	SPARQLstream [68]	SSN ontology	SPARQL query response	Continuous SPARQL query with time window	8000 data values/second	Yes	Cen	-	Data mapped to DSMS
Semantic Modelling	C-SPARQL [69]	RDF streams	SPARQL query response	Continuous SPARQL query (time window/ periodical execution)	-	Yes	Cen	-	Data mapped to DSMS No evaluation provided
Semantic Modelling	EP-SPARQL [70]	RDF and RDF event streams	SPARQL query response	Continuous SPARQL query	20,000 triples/20 locations	Yes	Cen	Prolog language	Designed for Event Processing
Semantic Modelling	CQELS [71]	RDF streams	SPARQL query response	Continuous SPARQL query	10 million triples	Yes	Dis	Java	Processing directly on Linked Stream Data
Semantic Modelling	LSM [27]	SSN ontology	SPARQL endpoint/HTTP REST	CQELS	70,000 sensor data sources	Yes	Cen	Java/Virtuoso/Hadoop	Integrated with XGSN [26] in OpenIoT [28]
Semantic Modelling	Q-ASSF [72]	SSN ontology	SPARQL query response	CQELS	200 queries/3000 sensors/16,000 triples	Yes	Cen	RabbitMQ/Apache Jena	Filtering algorithm to reduce sensor communications and triple transmission
Semantic Modelling	Linked Sensor Streams [44]	RDF streams	SPARQL query response	Type/Location based query (based on clustering)	20,000 stream data	Yes	Dis	Java	Combination of Linked Data and sensor streams

**Table 4:** Comparison of Search Techniques According to Content being Searched (Abbreviations are listed in the footer of Table 2).

Classification	Search Technique	Data Format	Access Approach	Search Type	Scale of Experiments	D^1^	Ar^2^	Implementation	Remarks
Instantaneous Data	LiveWeb [33]	Sensor data in XML	HTML/AJAX	Real-time content search based on keywords, category, and value range	-	Yes	Cen	PHP/Java/Apache/MySQL	Practical implementation without evaluation Should fit in global scale
Instantaneous Data	SensorMap [39]	Direct communication with web accessible sensors	Web services APIs	Spatial search (based on COLR-tree/crawling)	-	-	Cen	MSRSense toolkit	SenseWeb-based application, currently not accessible
Instantaneous Data	GeoCENS [40]	OGC SOS, SensorML, OGC O&M	SWE services	Spatial search (Peano space filling curves)	~40,000 sensors/procedures	Yes	P2P	-	Designed for sensor data from smaller organizations or individuals
Instantaneous Data	CQELS [71]	RDF streams	SPARQL query response	Continuous SPARQL query	10 million triples	Yes	Dis	Java	Processing performed directly on Linked Stream Data
Instantaneous Data Historical Data	IoT-SVKSearch [37]	Sampling values with metadata in Raw Data Store	Data in built-in format	Keyword-based (B^+^-tree), spatial-temporal (R-tree), value-based (B^+^-tree) search	140,800~352,000 sensors	Yes	Dec	PostgreSQL/file system	Multiple search functionalities supported
Instantaneous Data Historical Data	FUTS Data Query [42]	Virtual Object ontology in OWL-DL	SenML/JSON	Spatial search with time window/sensor type	20,000 sensors	Yes	Cen	Java	Cloud database for huge data storage
Historical Data	LSM [27]	SSN ontology	SPARQL endpoint/HTTP REST	CQELS	70,000 sensor data sources	Yes	Cen	Java/OpenLink Virtuoso/Apache Hadoop	Integrated with XGSN [26] in OpenIoT [28]
Context-based Sensor	OSIRIS [38]	OGC SWE Sensor model and data model	SWE services	Spatial, temporal and keyword-based search	-	Yes	Cen	JSI/Apache Lucene	Applications in Smart City domain, Website of the project no longer accessible
Context-based Sensor	Microsearch [31] Snoogle [32]	Direct communication with sensor motes with built-in metadata	Virtual sensors in built-in format	Keyword-based top-k search	-	Yes	Dis	TelosB motes	Early search implementations with limited functionalities and limited scalability
Context-based Sensor	Linked Sensor Data [53]	RDF	HTML/XML	SPARQL query	20,000 weather stations	Yes	Cen	OpenLink Virtuoso	Combination of Linked Data, sensors and sensor data
Context-based Sensor	SemSOS [56]	SWE based model in Linked Data	OGC SWE SOS	Named location based query	20,000 weather stations	No	Cen	RDF2Go/Sesame	Mobile sensors are ignored
Context-based Sensor	CASSARAM [73]	SSN ontology	SPARQL query response	SPARQL query	1,000,000 sensors	Yes	Dis	Apache Jena API	Multiple sensor feature support Fast top-k sensor selection
Context-based Sensor	VCS [74]	Server nodes (objects)	List of nodes	Keyword-based query with multiple features	-	Yes	P2P	-	No evaluation provided
Context-based Sensor	OpenIoT [28]	SSN ontology	SPARQL endpoint/HTTP REST	Continuous SPARQL query/Publish and Subscribe model	-	Yes	Dis	XGSN/LSM	Multiple practical applications provided
Context-based Sensor Context-based Entity	SPITFIRE [75]	Sensor (RDF triple)	SPARQL query response	CQELS	40 physical sensors	Yes	Cen	Jena Semantic Web Framework	Semantic sensor descriptions
Content-based Sensor	Fuzzy-based Sensor Search [76]	Sensor data streams	-	Sensor data stream based search for sensors	1500 data points/day	Yes	Dis	Java	Ranking based on sensor data prediction
Content-based Sensor Content-based Entity	Dyser [30]	Virtual sensors (microformat)	URL/HTTP/HTML	Keyword-based/value-based	385 sensors over 5 months	Yes	Cen	Java/PHP	Query of state of real world objects Computation required for state awareness, the scale is limited to small number of sensors
Content-based Sensor Content-based Entity	Sensor Ranking [77]	Sensor with outputs in built-in format	Ranked list of sensors	Prediction based ranking for content-based sensor search	20 sensors	Yes	Cen	C++	Local scale deployment
Context-based Entity	Gander [78]	Nodes (objects) (tuple space/tuple graph)	HTTP REST	Multi-hop query	20,000 mobile visitors	Yes	P2P	Java	Mobile applications for smart university
Context-based Entity	ISE [79]	Encrypted RFID records	XML/HTML/JSON	Database-based query	1,000,000 RFID records	Yes	Dis	Nginx/Tokyo Cabinet/C++	Cryptography for security
Content-based Entity	Correlation-based Sensor Search [80]	Sensor with sensor data in built-in format	List of sensors	Sensor search with a given state/output	384 sensors	Yes	Cen	Java/SMILE reasoning engine	Based on sensor data correlation
Context-based Entity	DiscoWoT [81]	Built-in description for resources (objects)	JSON/XML	RESTful interface search	-	Yes	Cen	AutoWoT toolkit	No evaluation provided

## Abbreviations

The following abbreviations are used in this manuscript:

AJAX	Asynchronous JavaScript and XML
APIs	Application Programming Interfaces
CoAP	Constrained Application Protocol
DNS	Domain Name System
DNS-SD	DNS Service Directory
DPWS	Devices Profile for Web Services
DSMS	Data Stream Management Systems
FUTS	Frequently Updated Timestamped and Structured
GSN	Global Sensor Networks
HTML	HyperText Markup Language
HTTP	Hypertext Transfer Protocol
IoT	Internet of Things
JSI	Java Spatial Index
JSON	JavaScript Object Notation
LSM	Linked Sensor Middleware
O&M	Observation and Measurement
O&M-S	Semantically annotated O&M
OGC	Open Geospatial Consortium
OWL	Web Ontology Language
OWL-DL	OWL- Description Logics
OWL-S	Semantic Markup for Web Services
P2P	Peer-to-Peer
RDB	Relational Database
RDB-to-RDF mapping language	R2RML language
RDF	Resource Description Framework
RDFS	RDF Schema
REST	REpresentational State Transfer
RFID	Radio-Frequency IDentification
SenML	Sensor Markup Language
SensorML	Sensor Model Language
SML-S	Semantically annotated SensorML
SOAP	Simple Object Access Protocol
SOS	Sensor Observation Service
SPARQL	SPARQL Protocol And RDF Query Language
SQL	Structured Query Language
SSN	Semantic Sensor Network
SSONs	Sensor Semantic Overlay Networks
SWE	Sensor Web Enablement
URIs	Uniform Resource Identifiers
URLs	Uniform Resource Locators
WADL	Web Application Description Language
WGS84	World Geodetic System 1984
WoT	Web of Things
XGSN	eXtended Global Sensor Networks
XML	eXtensible Markup Language
